# Seep-carbonate lamination controlled by cyclic particle flux

**DOI:** 10.1038/srep37439

**Published:** 2016-11-23

**Authors:** Tobias Himmler, Germain Bayon, David Wangner, Frieder Enzmann, Jörn Peckmann, Gerhard Bohrmann

**Affiliations:** 1 MARUM – Center for Marine and Environmental Sciences and Department of Geosciences, University of Bremen, 28334 Bremen, Germany; 2 IFREMER, Marine Geosciences Research Unit, Centre Bretagne, 29280 Plouzané, France; 3 Geological Survey of Denmark and Greenland, DK-1350 Copenhagen K, Denmark; 4 Institute of Geosciences, Johannes Gutenberg University Mainz, 55128 Mainz, Germany; 5 Institute of Geology, University of Hamburg, 20146 Hamburg, Germany; 6 Department for Geodynamics and Sedimentology, University of Vienna, 1090 Vienna, Austria

## Abstract

Authigenic carbonate build-ups develop at seafloor methane-seeps, where microbially mediated sulphate-dependent anaerobic oxidation of methane facilitates carbonate precipitation. Despite being valuable recorders of past methane seepage events, their role as archives of atmospheric processes has not been examined. Here we show that cyclic sedimentation pulses related to the Indian monsoon in concert with authigenic precipitation of methane-derived aragonite gave rise to a well-laminated carbonate build-up within the oxygen minimum zone off Pakistan (northern Arabian Sea). U–Th dating indicates that the build-up grew during past ~1,130 years, creating an exceptional high-resolution archive of the Indian monsoon system. Monsoon-controlled formation of seep-carbonates extends the known environmental processes recorded by seep-carbonates, revealing a new relationship between atmospheric and seafloor processes.

Carbonate build-ups develop at areas of seafloor methane venting where microbially mediated sulphate-dependent anaerobic oxidation of methane produces alkalinity, thus facilitating precipitation of seep-carbonates^1^,^2^,^3^,^4^. Seep-carbonates recently gained attention as geological archives, recording past methane release events^5^,^6^ and comprising significant sinks in the carbon cycle^7^,^8^. Despite being useful environmental archives, little is known about how atmospheric or astronomic processes influence seep-carbonate formation.

In the northern Arabian Sea, monsoon induced eutrophication in concert with sluggish inflow of low-oxygen water from the western Arabian Sea creates a stable water column oxygen minimum zone (OMZ; dissolved oxygen below 0.5 ml l^−1^ between ~100–1100 m water depth) along the Makran continental margin, off Pakistan^9^,^10^,^11^. The resulting hypoxic conditions on the seafloor are unfavourable for benthic organisms that would normally disrupt the sediment, allowing the preservation of varved sediments^12^,^13^. Yet, the Makran OMZ seafloor provides a habitat for microbial mats inhabiting seeps where methane and hydrogen sulphide emanate from the sea bed^14^,^15^. Although seasonal particle flux is known to generate laminated sediments in the sea bed of the OMZ^12^,^13^, its impact on local seeps and their chemosynthesis-based prokaryotes has not been explored to date. Previous studies have addressed environmental factors that impact carbonate formation at seeps in low-oxygen environments, but those studies were focused on seawater redox conditions^1^,^2^,^15^ and sulphate availability^16^. This study unravels the genesis of a seep-carbonate build-up form the Makran OMZ, demonstrating the impact of the Indian monsoon system on carbonate formation over the past ~1,130 years – a novel link between atmospheric processes and seafloor mineral authigenesis.

To gather evidence for the impact of seasonal sedimentation on the Makran OMZ seeps, a well-laminated, columnar authigenic carbonate build-up was examined. It is 47 cm long and varies between 12 and 17 cm in diameter, and was sampled from the seafloor in an area of active methane seepage (sample GeoB12353-11; N24° 48.44′, E63° 59.64′; 734 m water depth; Fig. 1). Such well-laminated carbonate build-ups have only been sampled within the Makran OMZ, whereas numerous methane seeps located in water depths below 1100 m are characterized by widespread carbonate crusts^15^. Carbonate fabrics were described macroscopically, by optical, and secondary electron microscopy (Fig. 2; see also Supplementary Fig. S1). Semi-quantitative X-ray diffraction, stable isotope analyses (δ^13^C, δ^18^O; Supplementary Table S1), and laser ablation-inductively coupled plasma-mass spectrometry (LA-ICP-MS) was used to reveal compositional variations (Fig. 2b,c,d; see Supplementary Table S2). Insights about structural properties were gained through X-ray computer tomography and results were used for a fluid flow simulation (Fig. 3; Supplementary Fig. S2). The timing of build-up growth was determined by U–Th dating, using an isochron approach (Table 1; Supplementary Fig. S3; e.g., refs 2, 16).

## Results and Discussion

### Structural build-up properties and dominant carbon source

**Figure 1:**
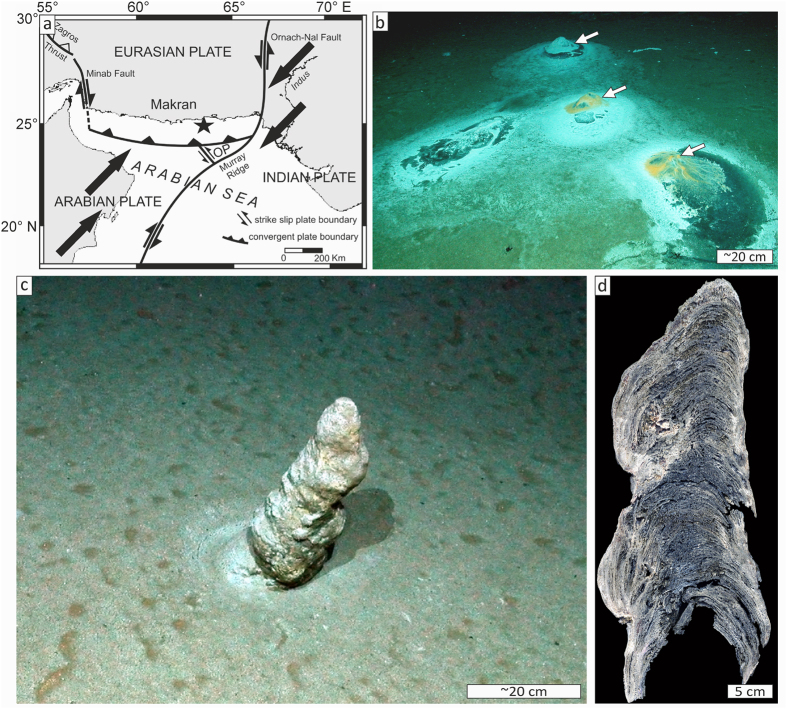
Sampling location and macroscopic characteristics of the carbonate build-up. (**a**) Tectonic sketch map of the Makran convergent margin (redrawn after ref. 36); asterisk indicates sample location (OP = Omara Plate); arrows indicate the general wind direction during summer (SW) and winter (NE) monsoon. (**b**) ROV seafloor image (dive 196) showing four active gas emission sites covered by white and orange patches of microbial mats at the sampling site; arrows point out the domal geometry of bacterial mats caused by carbonate growth below the mats; note the colour difference between black sediment underneath the microbial mats and the surrounding olive-grey seafloor. (**c**) ROV photograph of the studied columnar carbonate build-up which was not covered by a bacterial mat as the other active methane emission sites (dive 196; sample GeoB12353-11). (**d**) Colour scan of polished slab revealing internal lamination (reflected light). Scales in (**b**,**c**) are approximate.

**Figure 2:**
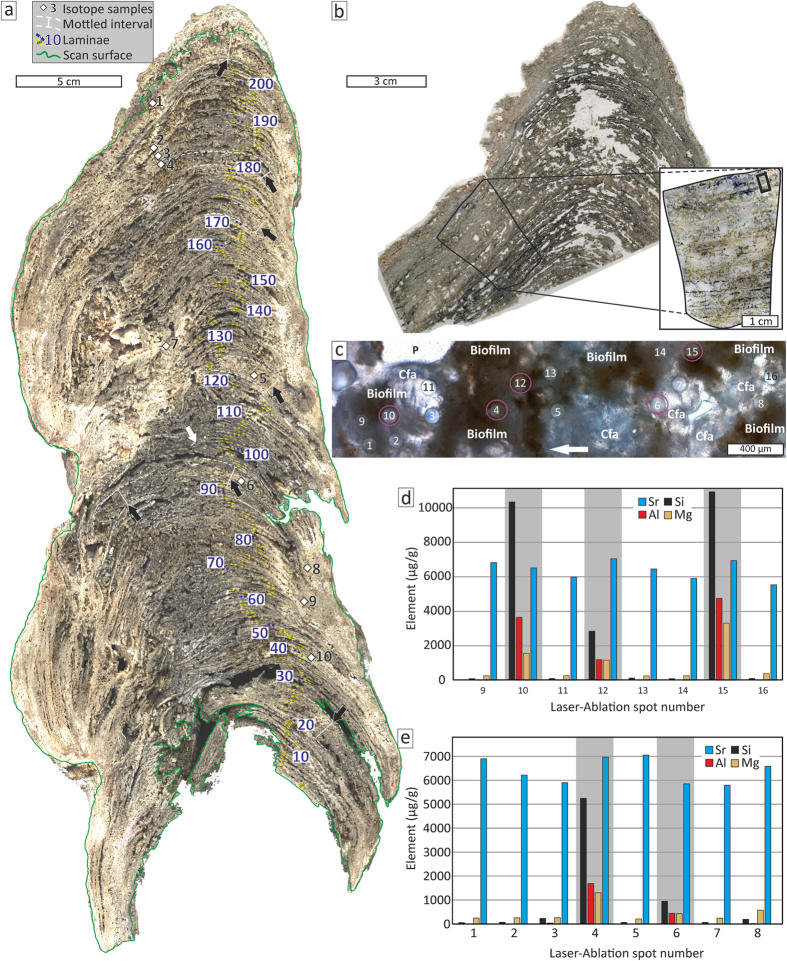
Laminae counting and elemental composition analysed with LA-ICP-MS. (**a**) Scan of epoxy impregnated, polished slab, highlighting individual 204 laminae and spots of isotope samples (1–10; see Supplementary Table S1); note mottled intervals (black arrows) sometimes lack distinct lamination; white arrow indicate overlapping of lamina #101; epoxy resin and unfilled porosity appear dark grey to black. (**b**) Thin-section scan of the build-up top displaying the relative position of the thin section used for LA-ICP-MS (inlet) and the actual ablation spots (black rectangle in the upper right of the blown-up section). (**c**) Thin section micrograph (plane-polarized light) displaying the LA-ICP-MS ablation craters within clotted and fibrous aragonite (Cfa) and fossilised biofilms (see also Supplementary Fig. S1); craters that yielded high aluminium (Al) contents are associated with biofilms (purple circles); arrow points towards the top. (**d**,**e**) bar diagrams showing element contents of LA-ICP-MS analyses; high strontium (Sr) contents agree with aragonite as dominant carbonate cement; spots with relative high contents of terrigenous aluminium (Al) are highlighted (grey bands); note that high silicon (Si) and magnesium (Mg) contents correlate with high Al contents.

**Figure 3:**
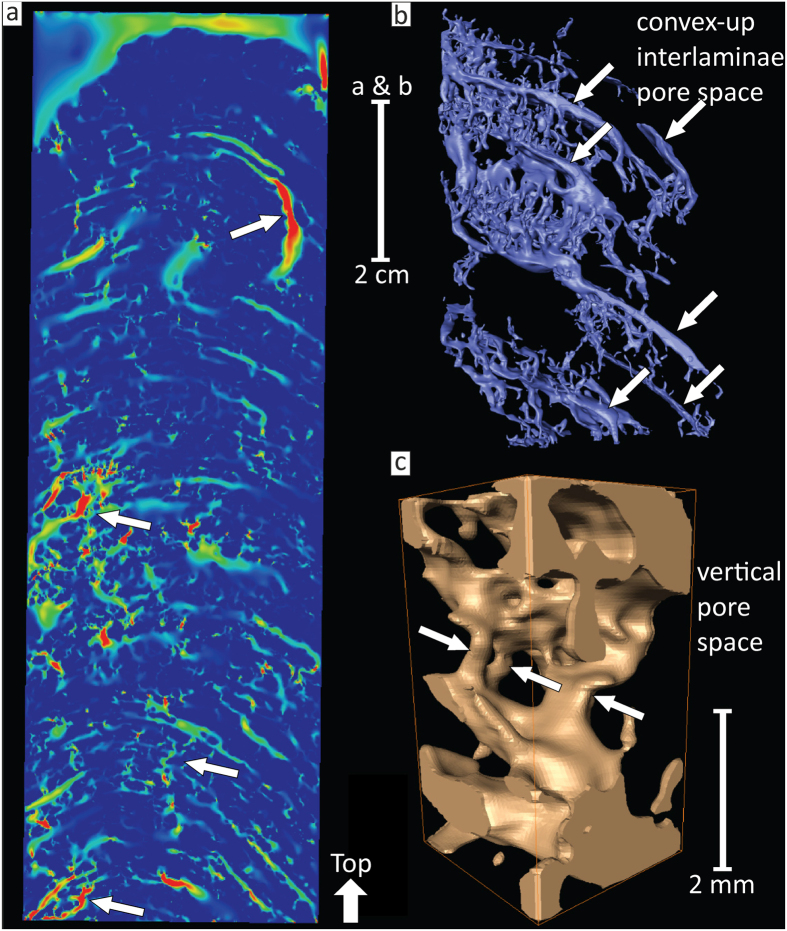
Simulation of gaseous methane flow through the build-up. (**a**) 2D still frame of the 3D fluid flow simulation showing relatively high (red) flow velocities (arrows) versus relatively low to zero velocities (green to blue; for details see Supplementary Tables S4). (**b**) Image of the 3D pore space network displaying convex-up interlaminae pore space (arrows). (**c**) Close-up of vertical pores (arrows) connecting two convex-up interlaminae pores.

Numerous millimetre to submillimetre thin convex-up shaped laminae comprise the build-up framework, overall displaying a stromatolitic fabric. Laminae are internally consistent and generally less than 1 mm thick, except for centimetre scale mottled intervals in the middle and top part (Fig. 2a). A total of 204 laminae were counted visually; considering the mottled intervals and potential underestimation due to the limited optical resolution, a 10% error margin is applied to the visual counts (i.e. 204 ± 20). The framework is mainly composed of ^13^C-depleted fibrous and clotted aragonite cement (Figs 1d and 2a,b,c; Supplementary Table S1). Exclusively negative δ^13^C_aragonite_ values (−55.1 to −41.3‰ VPDB) point to seepage methane (δ^13^C_methane_ = −70‰ VPDB; ref. 15) as the dominant carbon source and agree with sulphate-dependent anaerobic oxidation of methane (AOM: CH_4_ + SO_4_^2−^ → HCO_3_^−^ + HS^−^ + H_2_O; e.g., ref. 17) – the biogeochemical key process inducing carbonate precipitation at methane seeps (e.g., refs 1, 2, 3, 4). Inside the build-up, upward flow of gaseous methane was facilitated by the porous carbonate framework (permeability ~1.088 * 10^−9^ m^2^; porosity between 5–16 vol.%; Fig. 3; Supplementary Fig. S2).

### Timing of build-up growth

The temporal context for build-up growth is provided by mean U–Th isochron ages which were obtained for the lower (1.13 ± 0.28 ka), central (0.14 ± 0.04 ka), and top part (0.07 ± 0.02 ka; Table 1). Despite the uncertainties inherent to U–Th dating of seep-carbonates^18^, the ages indicate relatively fast growth (i.e. ~470 mm ka^−1^). Theoretical simulation of seep-carbonate growth showed that the development of a 10–20 mm thick carbonate crust takes about 310 years^4^ (i.e. approximately 48 mm ka^−1^). A field study yielded similar values (~50 mm ka^−1^) for methane-derived aragonite^18^. Apparently, the Makran build-up had grown about one order of magnitude faster. Fast growth also agrees with, and was favoured by the high sedimentation rate for the Makran OMZ (~1.2 mm year^−1^)^12^. If the sedimentation rate would have exceeded build-up growth, the structure would have been buried with time^4^. Before methane seepage, and hence carbonate accretion, had ceased for an unknown reason ~70 (±20) years ago, the build-up had grown above the sediment–water interface.

Seep-carbonate build-ups in the Black Sea basin and in the eastern Mediterranean Sea have grown in a similar fashion in oxygen-restricted environments, causing them to project above the sediment–water interface^1^,^2^. However, none of the previously described build-ups show regular lamination indicative of a cyclic formation mode. Given that the Makran build-up grew into the water column, AOM-induced carbonate accretion was modified by seasonal sedimentation events, which are common in the Arabian Sea^11^,^12^,^19^. Assuming constant methane flow and AOM-induced carbonate precipitation, build-up lamination was likely controlled by cyclic sedimentation events. The reported sedimentation peaks, according to monsoonal particle flux^11^,^12^, suggests that two laminae formed per year (i.e. the 204 ± 20 laminae were formed during ~102 years). A growth period of ~102 years is, however, is stark contrast with U–Th ages in the lower and middle portions, which indicate much slower growth (Table 1).

Assuming constant growth without periods of seepage quiescence, two distinct growth phases can be identified based on the isochron U–Th ages: The lower portion comprises about 102 (±10) laminae that formed between 1.13 ± 0.28 and 0.14 ± 0.04 ka, corresponding to a growth rate of approximately one lamina every ten years (~1000 years/~102 laminae). In contrast, the remaining 102 laminae formed between 0.14 ± 0.04 and 0.07 ± 0.02 ka, equivalent to approximately one lamina per year (72 years/102 laminae). This indicates a marked increase of build-up growth rate. Alternatively, build-up growth may not have been constant due to periods of seepage quiescence and periods of methane seepage fed from reservoirs below. Changes in methane flux are known from pore water data of sediment cores at the Makran margin, and are potentially triggered by earthquake shaking of the seafloor^20^. Such changes in growth rates, however, cannot be unravelled on the basis of the three isochron ages reported here.

### Origin of lamination

The laminated Makran OMZ sediments have been shown to reflect a wide range of different cyclicities, ranging from <10 to 750 years^12^,^21^. Interestingly, high-frequency cycles (<10 years) have been interpreted to reflect monsoonal sedimentation events^12^, which can be overprinted by lunar tide effects^21^. It is therefore likely that the build-up lamination reflects a combination of both, tidal and seasonal forcing, with the latter one becoming more prominent for build-up growth since at least 0.14 ka. It is noteworthy that a lacustrine stromatolite, which was not affected by monsoonal circulation, was found to reveal a similar growth periodicity of one laminae per four to six years over a growth period of ~1000 years^22^. This lamination pattern was interpreted to reflect mainly climate forcing linked to Pacific sea surface temperature and El Niño–Southern Oscillation (ENSO) dynamics^22^. However, the influence of ENSO dynamics on sedimentation patterns of the Makran OMZ was rendered insignificant^12^,^19^ and its influence on the Indian monsoon is not straightforward^23^. We therefore argue that ENSO was not a major factor for the cyclic growth of the build-up, but put forward that the observed cyclicity reflects monsoonal sedimentation patterns.

Coverage by microbial mats or biofilms is considered significant for seep-carbonate build-up formation above the sediment surface. Whereas methane oxidation with oxygen (CH_4_ + 2 O_2_ → CO_2_ + 2 H_2_O) inhibits carbonate precipitation^24^, reducing conditions beneath mats of sulphide oxidizing bacteria supported AOM even in oxygenated waters on the Cascadian margin^25^. Moreover, microbial mats baffle and bind settling particles, thereby contributing to build-up lamination. The main build-up component, fibrous AOM-induced aragonite, is locally covered by brownish organic matter, which is interpreted as remains of microbial biofilms and mats (see Supplementary Fig. S1). LA-ICP-MS analyses revealed variations in elemental compositions between the clear, pure variety of aragonite, and biofilm-covered aragonite (Fig. 2c,d,e; Supplementary Table S2): In contrast to pure aragonite, biofilm-associated aragonite yielded higher silicon, aluminium, magnesium, titanium, and zirconium contents, the latter two being indicative of terrigenous sediment on the Makran continental margin^26^. Similarly, yttrium to holmium ratios of biofilm-associated aragonite (14 < Y/Ho < 30) are similar to upper continental crust material (Y/Ho ≈ 27), but significantly lower than seawater (47 < Y/Ho < 77; e.g., ref. 27) and lower than the ratios found for pure aragonite (49 < Y/Ho < 217).

Although the provenance of terrigenous sediment cannot be deduced from the LA-ICP-MS data alone, it likely derived from the Makran hinterland. During winter (NE) monsoons, enhanced river runoff transports loads of suspended sediment onto the shelf. Subsequently, terrigenous fines are transported farther down-slope by agitation and redeposition^12^,^19^. Remarkably, winter monsoon irrigation in the Makran hinterland increased within the past 1,000 years^28^, possibly impacting build-up growth within the OMZ. The baffling and binding effect of microbial mats covering the build-up is also apparent from biogenic fines on laminae surfaces. The most abundant constituents were coccoliths of surface-water dwelling calcareous nannoplankton species, including *Gephyrocapsa oceanica, Emiliania huxleyi, Calcidiscus leptoporus, Umbilicosphaera sibogae* (Supplementary Fig. S1). Whereas *E. huxleyi* is a cosmopolitan species, the other abundant species (*G. oceanica, C. leptoporus*, and *U. sibogae*) represent an assemblage typical for nutrient rich surface water conditions during the monsoon^29^. It needs to be stressed that the varved pattern described for Makran OMZ sediments^12^,^13^,^19^,^21^ is not observed within the examined build-up. The OMZ sediments comprise alternations of dark (i.e. rich in organic matter and coccoliths, interpreted as winter monsoon deposit) and light layers, interpreted as summer monsoon deposits (rich in terrigenous material). In contrast, the light-dark lamination of the build-up comprises pure authigenic aragonite cement, and aragonite partly covered with biofilm remains that bound and baffled settling particles (Fig. 2; Supplementary Fig. S1). Consequently, an attribution of summer or winter monsoon particles based on the compositional data alone remains ambiguous despite the evident influence of particle flux on laminae formation.

## Conclusions

In summary, this study reveals that the lamination of a methane-derived carbonate build-up was mainly controlled by cyclic sediment influx. Age constraints on build-up growth indicate that the Indian monsoon possibly in combination with tidal forcing gave rise to the delicate lamination, in concert with carbonate precipitation induced by anaerobic oxidation of methane. This study provides compelling evidence for the influence of the global weather and astronomical forcing on seep-carbonate formation – a previously unrecognized relationship. In particular, the Indian monsoon is a crucial parameter that steers the formation of laminated carbonates at seeps on the Makran continental margin within the OMZ.

## Methods

### Seafloor sampling, splitting, and colour scanning

The sample was taken from the seafloor using the manipulator arm of the ROV MARUM-QUEST 4000 m during R/V METEOR expedition M74/3 in 2007^30^. Once on board, the sample was dried and stored for further studies onshore. The build-up was cut in two halves using a wet-cutting rock saw. From one half a ~2 cm thick slab was cut off and embedded in epoxy resin in order to preserve the internal fabric. The slab surface was polished and subsequently scanned with a smartCIS 1600 Line Scanner equipped with a Triple Line Scan Camera SK12240GKOC-LB at 500 dpi resolution. One half was used to prepare subsamples for thin sections and high-resolution X-ray computer tomography (HRXCT).

### Mineralogy, petrography, and stable isotope analysis

Semi-quantitative X-ray diffraction analysis was performed on one bulk-rock powder sample obtained with a handheld micro drill from the cut surface, using a Phillips X’Pert Pro MD X-ray diffractometer equipped with a Cu-Kα-tube (λ = 1.541; 45 kV, 40 mA). Large thin sections (100 × 150 mm) were prepared from cut slabs and examined using standard petrographic microscopy on a Zeiss Axioskop 40 A microscope. Carbonate carbon and oxygen stable isotopes were analysed on powders reacted with 100% phosphoric acid at 75 °C. The released carbon dioxide was injected into a Finigan MAT 251 mass spectrometer. Carbonate δ^13^C and δ^18^O values are reported in per mill (‰) relative to the Vienna PeeDee Belemnite (VPDB) standard. Reproducibility was checked by replicates of an internal standard calibrated against the NBS19 standard. Long term standard deviation for carbonate δ^13^C and δ^18^O analyses is less than 0.05‰ (δ^13^C) and 0.07‰ (δ^18^O).

### Digital and scanning electron microscopy

Subsamples of individual laminae were taken using a stainless steel tweezer and photographed with a VHX-5000 digital microscope under reflected light at ZMT (Bremen, Germany). The surface of individual laminae was scanned with a ZEISS field emission scanning electron microscope (University of Bremen, Germany) at 3.0 kV beam current and ~5 mm scanning distance.

### Thin section laser ablation inductively coupled plasma mass spectrometry

Laser ablation analyses were carried out at the Geoscience Department, University of Bremen, using a NewWave UP193 laser connected in line to a ThermoFinigan Element2 inductively coupled plasma mass spectrometer. The laser was operated at a 5 Hz pulse rate at approximately 1.3 GW/cm^−2^ irradiance, using a beam diameter of 100 μm. Samples were analysed for 90 seconds, using low mass resolution mode. For each spot analyses, a pre-ablation step with 120 μm beam diameter (approximately 0.3 GW/cm^−2^, 1 Hz) was done to avoid surface contamination. Data were calibrated against the NIST610 glass standard with contents reported by^31^. Element contents were quantified with the Cetac GeoPro software, using ^43^Ca as internal standard and assuming 40.00 wt.-% Ca content of the analysed aragonite cement. Because of the high Sr content of the sample, the interference of ^86^Sr^2+^ on ^43^Ca^+^ + ^43^Ca^+^ was corrected using a factor derived from one measurement of a Sr-rich carbonate standard (MACS-3; data not shown; see ref. 32). Data quality was assessed by analysing USGS reference materials BCR2G and BHVO2G (basaltic glasses) along with the samples; the data are summarized in Supplementary Table S3.

### Analytical procedures for U-Th measurements and isochron dating

Fragments of individual layers were sampled with a stainless steel tweezer and pulverized using agate mortar and pestle. Between 14 and 93 mg of material was dissolved in 7.5 NHO_3_ before adding 200 μl of a mixed ^236^U/^229^Th spike and subsequent digestion in concentrated HNO_3_ on a hot plate. Dissolved U and Th were concentrated using Fe-oxyhydroxide co-precipitation and isolated by ion exchange chromatography. Uranium and Th concentrations and isotopes were analysed with the Neptune multi collector-inductively coupled plasma-mass spectrometer at the Pôle Spectrométrie Océan, IFREMER. Measured isotope ratios were corrected for mass discrimination by standard bracketing, using IRMM-184 (U) and IRMM-035 (Th) standard solutions. Precision of ^238^U/^234^U and ^229^Th/^230^Th measurements was generally better than 50‰ and 5‰, respectively. Apparently co-genetic laminae were dated (Table 1) with an isochron approach; ages were calculated in three dimensional mode using the Isoplot software (Version 3.75; ref. 33). Seep-carbonates contain variable amounts of detrital material (e.g., clay minerals), representing significant inherited ^230^Th (detrital fraction) in addition to the ^230^Th (authigenic fraction) from uranium decay within the carbonate lattice. The application of isochron dating methods helps circumvent this problem. Corrected isochron ages can be calculated by analysing multiple sub-samples of the same age, comprising various proportions of carbonate and detrital fractions. It was assumed that the sub-samples collected from the upper (n = 3), middle (n = 2), and lower (n = 2) parts of the build-up are co-genetic, and were thus used to calculate distinct average isochron ages for each part. The measured activity ratios (^232^Th/^238^U, ^230^Th/^238^U, and ^234^U/^238^U) and the nuclide half-lives (^238^U, ^234^U, ^232^Th, and ^230^Th) of ref. 34 were used for calculations. Individual sample ^230^Th/U ages were obtained with the 230Th/U Isoplot spreadsheet function (Table 1). Because the error of the isochron age using all activity ratios are unrealistically small, an error margin of ±25% was applied which corresponds to the external U–Th isotopic measurement reproducibility obtained from repeated analyses of an in-house seep-carbonate standard (see refs 2, 35).

### Quantitative porosity measurements

Before cutting the build-up it was scanned using the medical X-ray computer tomography (CT) facility at the hospital Klinikum Bremen Mitte (Bremen, Germany). The X-ray source voltage was 120 kV at a current of 600 mA. Scan resolution was 1 mm in x–y–z directions. The CT data were processed with the Amira software (VGS, Burlington, U.S.A.). Two materials, i.e. pore space and carbonate, were defined by using the Amira semiautomatic grey-scale segmentation editor. Porosity was quantified for each CT-scan by calculating the percentile area of pore space per tomographic slice.

### High-resolution CT scan and gas flow simulation

A representative subsample was cut from the epoxy-resin embedded working half for high-resolution X-ray computer tomography (HRXCT) scanning with the CT-Alpha scanner (ProCon X-ray, Garbsen, Germany) at the Institute of Geosciences, Johannes-Gutenberg-University Mainz, Germany. The device is equipped with a micro focus X-ray tube featuring a diamond coated target and a 2048*2048 pixels detector. A three dimensional tomogram was assembled from multiple 2D projections with the Amira software. Based on the 3D tomograms the gas flow through a representative segment from the upper central part of the build-up towards the top was simulated using the GeoDict software. The permeability for the segment was 1.088*10^−9^ m^2^. Open pore space was defined empty (permeable) and carbonate layers as filled space (impermeable). Pore filling material was characterized with the properties shown in Supplementary Table S4. Gas inflow was limited to the bottom part of the simulation, applying an external overpressure of 0.35 bar. Outflow towards the sides from open pores was blocked by digitally coating the outer surface with impermeable material (=filled pore space).

## Additional Information

**How to cite this article**: Himmler, T. *et al*. Seep-carbonate lamination controlled by cyclic particle flux. *Sci. Rep.* **6**, 37439; doi: 10.1038/srep37439 (2016).

**Publisher's note:** Springer Nature remains neutral with regard to jurisdictional claims in published maps and institutional affiliations.

## Supplementary Material

Supplementary Information

## Figures and Tables

**Table 1:** U–Th isotopic data (±2σ) and activity ratios (^232^Th/^238^U), (^230^Th/^238^U) and (^234^U/^238^U) used for isochron age calculations (see also Supplementary Fig. S3); activity ratios were corrected for mass fractionation and spike contributions; n. d. = not determined; δ^234^U_(0)_ represents the measured activity ratios at time zero (today) expressed in delta notation (δ^234^U = [((^234^U/^238^U) − 1) ∗ 1,000]).

Sample	Weight (mg)	^238^U (μg/g)	^232^Th (μg/g)	^230^Th (pg/g)	^230^Th/^232^Th	Activity ratios			δ^234^U_(0)_ (‰)	^230^Th/U age* (ka)	Isochron age** (ka)
						(^232^Th/^238^U)	(^230^Th/^238^U)	(^234^U/^238^U)			
1	15.00	2.16 ± 0.01	0.234 ± 0.001	0.921 ± 0.07	3.93	0.035 ± 0.001	0.026 ± 0.002	1.149 ± 0.002	149 ± 1	0.46 ± 4	
2	93.44	7.63 ± 0.01	1.209 ± 0.002	4.032 ± 0.024	3.34	0.052 ± 0.001	0.032 ± 0.001	1.125 ± 0.001	125 ± 1	0.13 ± 5	
1&2											1.13 ± 0.28
3	59.84	2.81 ± 0.01	1.103 ± 0.008	3.59 ± 0.12	3.25	0.129 ± 0.001	0.078 ± 0.003	1.128 ± 0.002	128 ± 1	0.13 ± 12	
4	25.26	3.20 ± 0.01	0.119 ± 0.001	0.46 ± 0.01	3.85	0.012 ± 0.001	0.009 ± 0.001	1.140 ± 0.003	140 ± 3	0.14 ± 1	
3&4											0.14 ± 0.04
5	13.77	3.81 ± 0.03	0.172 ± 0.001	0.68 ± 0.02	3.98	0.015 ± 0.001	0.011 ± 0.001	1.151 ± 0.009	151 ± 8	0.20 ± 2	
6	39.56	29.9 ± 2.3	0.785 ± 0.004	2.51 ± 0.05	3.20	0.009 ± 0.001	0.005 ± 0.001	1.136 ± 0.003	136 ± 3	n. d.	
7	24.80	0.974 ± 0.002	0.199 ± 0.001	0.71 ± 0.02	3.55	0.067 ± 0.001	0.044 ± 0.001	1.145 ± 0.004	145 ± 4	0.42 ± 6	
5&6&7											0.07 ± 0.02

^*^^230^Th/U ages were calculated using the Th230Age spreadsheet function of Isoplot.

^**^Isochron ages were derived from Osmond isochrons calculated as linear 3-dimensional projection, using the measured activity ratios.
