# Supplementary material for: Seep-carbonate lamination controlled by cyclic particle flux
Source: Sci Rep. 2016 Nov 23;6:37439. doi: 10.1038/srep37439 (PMC5120270; doi:10.1038/srep37439)
Supplement: Supplementary Information [file srep37439-s1.pdf]

Seep-carbonate lamination controlled by cyclic particle flux

Tobias Himmler[1,2,*], Germain Bayon[2], David Wangner[1,3], Frieder Enzmann[4], Jörn Peckmann[5,6] &

Gerhard Bohrmann[1]

[1]*MARUM – Center for Marine and Environmental Sciences and Department of Geosciences, University of Bremen, 28334 Bremen, Germany*
[2]*IFREMER, Marine Geosciences Research Unit, Centre Bretagne, 29280 Plouzané, France*
[3]*Geological Survey of Denmark and Greenland, DK-1350 Copenhagen K, Denmark*
[4]*Institute of Geosciences, Johannes Gutenberg University Mainz, 55128 Mainz, Germany*
[5]*Institute of Geology, University of Hamburg, 20146 Hamburg, Germany*
[6]*Department for Geodynamics and Sedimentology, University of Vienna, 1090 Vienna, Austria*

*Corresponding author (thimmler[at]uni-bremen.de)

Content

I. Supplementary figures

II. Supplementary tables

III. SEM-EDX spectrum

I. Supplementary figures

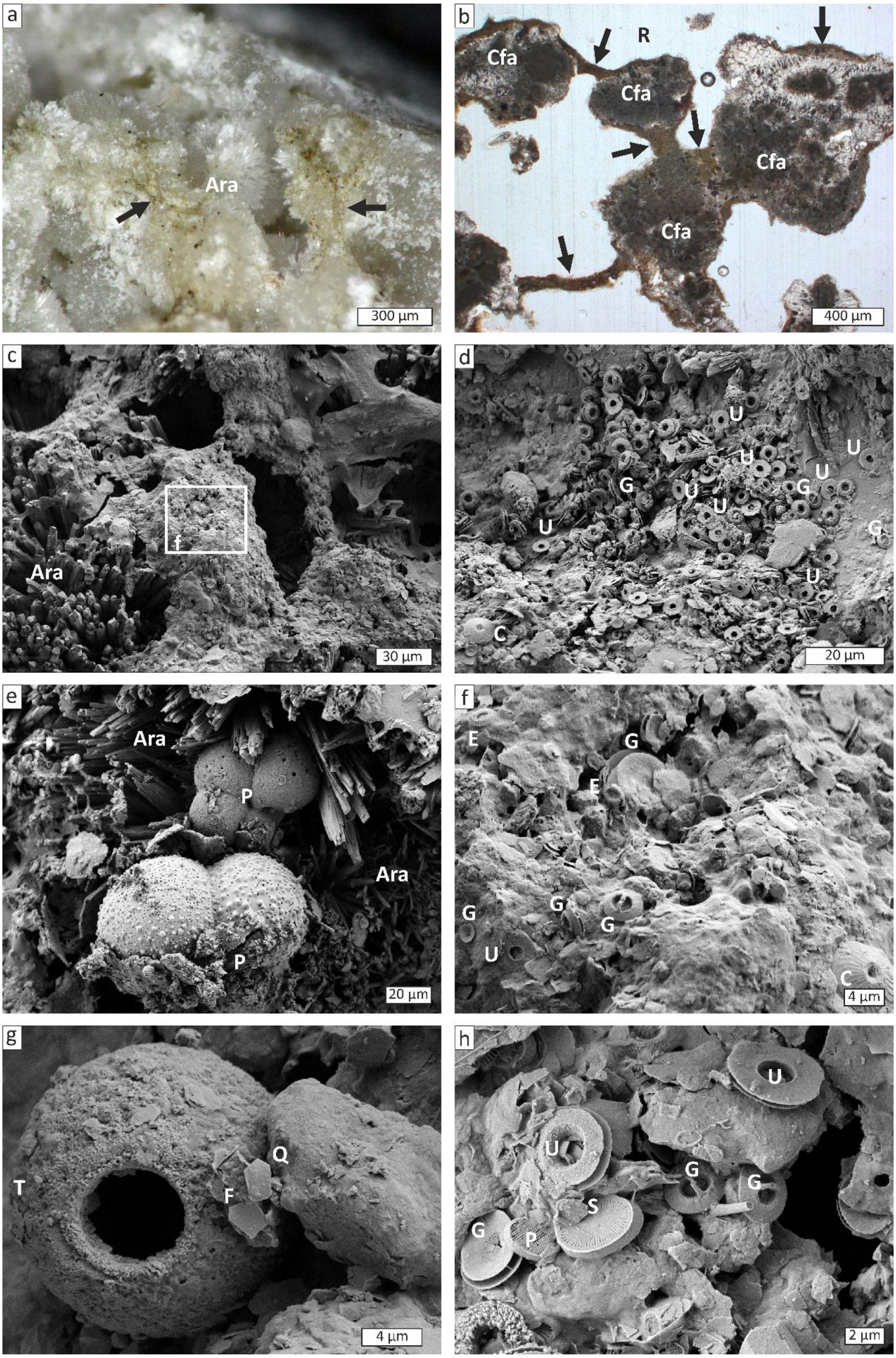

Figure S1: **Optical (a, b) and scanning electron microscope images of laminae surfaces (see Supplementary Fig. 3 for sample locations).**

(**a**), Fibrous aragonite (Ara) cement partly covered with remains of a brownish biofilm; note connections between biofilm patches (arrows; reflected light; sample He-6-11). (**b**), Thin-section micrograph displaying biofilm remains (arrows) bridging resin impregnated (R) pore space between clotted and fibrous aragonite (Cfa) aggregates (plane-polarized light). (**c**), Biofilm with abundant coccoliths covering fibrous aragonite crystals (Ara; sample He-6-10); white rectangle corresponds to area shown in (**f**). (**d**), Laminae surface (sample He-11-3) with coccolith assemblage of *Umbilicosphaera sibogae* (U), *Gephyrocapsa oceanica* (G), and *Calcidiscus leptoporus* (C). (**e**), Planktonic formanifera (P) tests embedded in fibrous aragonite (Ara; sample He-11-6). (**f**), Magnification of laminae surface shown in (**c**), highlighting abundant coccoliths (*U. sibogae*, *G. oceanic*, *C. leptoporus*, and *Emiliania huxleyi* (E)). (**g**), Calcareous dinoflaggelate *Thoracosphaera heimii* (?) (T), coccoliths of *Florisphaera profunda* (F), and a quartz grain (Q; relatively high Si and O counts during energy dispersive X-ray analyses; see below; sample He-6-13). (**h**), Assemblage of *U. sibogae*, *G. oceanica*, and the holococcoliths *Syracosphaera pulchra* (S), and *Calyptrolithophora papillifera* (P; sample He-11-2).

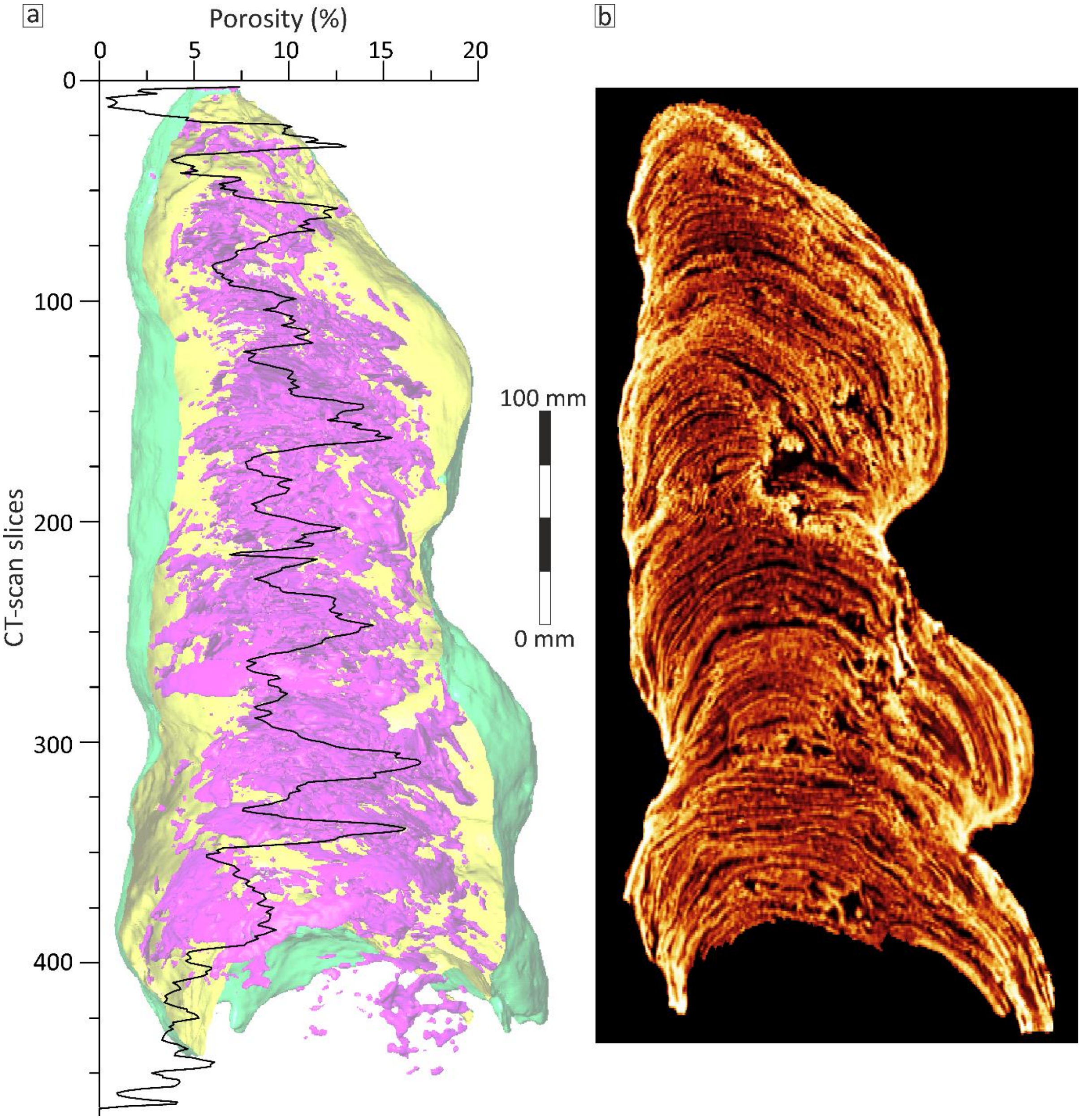

Figure S2: Porosity measurements and computer tomography (CT).

(**a**), Line plot of CT-based porosity measurements overlying a 3D model of the build-up; pore space is developed best in the central portion and shown in purple; note, carbonate material is not displayed; yellow represent the inside surface and green the exterior surface. (**b**), 2D tomogram highlighting the fine carbonate laminae shown in orange and the pore space (black).

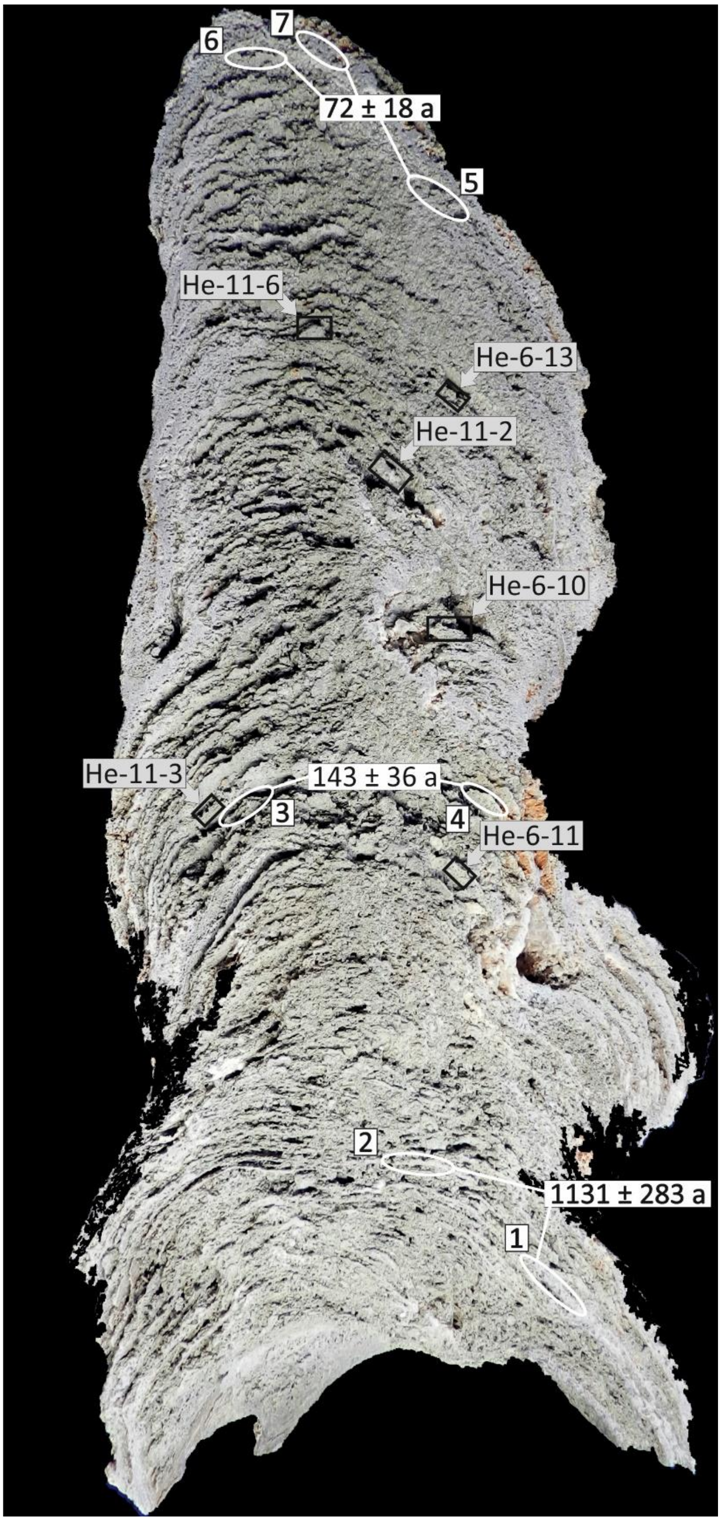

Figure S3: Locations of U–Th and SEM samples.

White ovals indicate locations of subsamples (1 to 7) used for U–Th isochron age calculations; respective ages are shown in white boxes (see Table 1 for details); black rectangles highlight SEM samples shown in Supplementary Fig. S1; respective sample labels are shown in grey boxes (He-6-10, He-6-11, He-6-13, He-11-2, He-11-3, and He-11-6).

II. Supplementary tables

Table S1: Carbon and oxygen stable isotope compositions.

$\delta^{13}C = [((^{13}C/^{12}C)_{sample}/(^{13}C/^{12}C)_{standard})-1]*1{,}000$; $\delta^{18}O = [((^{18}O/^{16}O)_{sample}/(^{18}O/^{16}O)_{standard})-1]*1{,}000$; for sample location see Fig. 2a in the main text; A.V. = average value; S.D. = standard deviation. The $\delta^{18}O_{fluid}$ is given in per mil vs. Standard Mean Ocean Water (SMOW) and was calculated after Han et al. (2004): $\delta^{18}O_{fluid}$ (SMOW) = $\delta^{18}O_{aragonite}$ (VPDB)–((19.7–T)/4.34)), with temperature T = 10.65 °C (measured bottom water temperature during sampling; see ref. 15).

Sample	$\delta^{13}C_{carbonate}$ (VPDB)	$\delta^{18}O_{carbonate}$ (VPDB)	$\delta^{18}O_{fluid}$ (SMOW)
1	−41.3	1.8	−0.30
2	−50.4	2.2	0.14
3	−51.6	2.2	0.10
4	−55.1	2.4	0.31
5	−46.1	1.9	−0.16
6	−52.0	2.1	0.04
7	−51.7	2.4	0.31
8	−54.8	2.3	0.18
9	−49.3	2.3	0.17
10	−54.6	2.2	0.08
A.V. ± S.D.	−50.7 ± 4.1	2.2 ± 0.2	0.09 ± 0.18

Table S2: Major and trace element contents (µg/g) measured with LA-ICP-MS. See Fig. 2 for sample location ; Cfa = clotted and fibrous aragonite; n.d. = not detected; n.c. = not calculated.

Spot number	Lithology	Si	Al	Mg	Sr	Ti	Zr	Y/Ho
1	Cfa	55.7	1.09	235	6,890	0.141	0.020	n.c.
2	Cfa	68.0	0.595	247	6,214	0.188	0.012	n.c.
3	Cfa	227	34.36	255	5,894	1.63	0.026	64
4	Cfa +biofilm	5,238	1,659	1,271	6,953	59.4	1.54	14
5	Cfa	66.9	0.632	203	7,046	0.196	0.013	121
6	Cfa +biofilm	941	438	419	5,844	15.3	0.719	23
7	Cfa	63.5	0.251	237	5,912	0.184	0.008	n.c.
8	Cfa	193	16.1	572	6,713	0.571	0.039	161
9	Cfa	65.5	2.13	257	6,848	0.254	0.015	49
10	Cfa +biofilm	10,369	3,656	1,544	6,542	268	3.74	27
11	Cfa	73.1	5.15	273	6,005	0.457	0.020	60
12	Cfa +biofilm	2,828	1,200	1,153	7,069	50.3	0.960	18
13	Cfa	96.2	10.2	256	6,506	0.333	0.012	n.c.
14	Cfa	67.8	n.d.	267	5,954	0.048	0.006	n.c.
15	Cfa +biofilm	11,034	4,767	3,288	6,995	207	5.17	26
16	Cfa	75.1	1.12	387	5,583	0.288	0.012	217

Table S3: Average element contents of standard replicates (BCR2G, BHVO2G) measured during LA-ICP-MS analyses. Respective reference data from GeoReM data base (MPI Mainz, http://georem.mpch-mainz.gwdg.de, accessed March 2016).

Element	BCR2G (n=2)		BHVO2G (n=2)	
	This study (µg/g)	GeoReM (µg/g ± 2σ)	This study (µg/g)	GeoReM (µg/g ± 2σ)
Mg	19,179 ± 1,300	21,467 ± 543	40,888 ± 2,434	42,994 ± 121
Al	72,300 ± 8,664	70,926 ± 2,117	76,068 ± 7,921	71,985 ± 529
Si	246,872 ± 7,088	253,867 ± 55,241	237,902 ± 7,498	230,428 ± 467
Ti	12,360 ± 598	14,100 ± 1,000	15,763 ± 808	16,300 ± 900
Sr	326 ± 11	342 ± 4	396 ±10	396 ± 1
Zr	170 ± 2	184 ± 15	161 ± 2	170 ± 7
Y	32 ± 1	35 ± 3	24 ± 0.1	26 ± 2
Ho	1.23 ± 0.17	1.72 ± 0.08	0.91 ± 0.12	0.98 ± 0.04

Table S4: Parameters used for the fluid flow simulation with GeoDict.

Parameter	Unit
Medium	Methane
Phase	Gas
Fluid pressure	77 bar
Temperature	4 °C
Density	64.360308 [kg/m^3]
Dynamic viscosity	12.6558 [10^{-6} Pa s]

III. <u>SEM-EDX spectrum (sample He-6-13)</u>

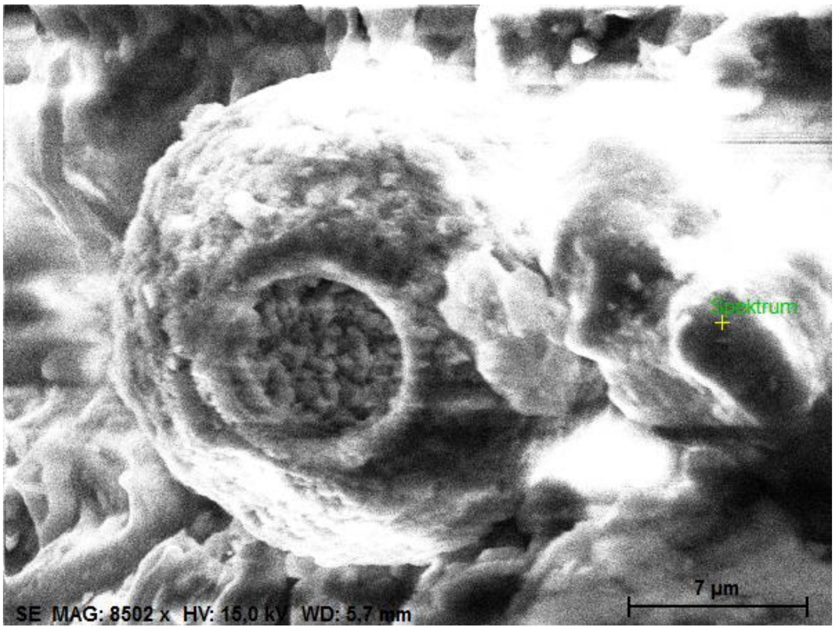

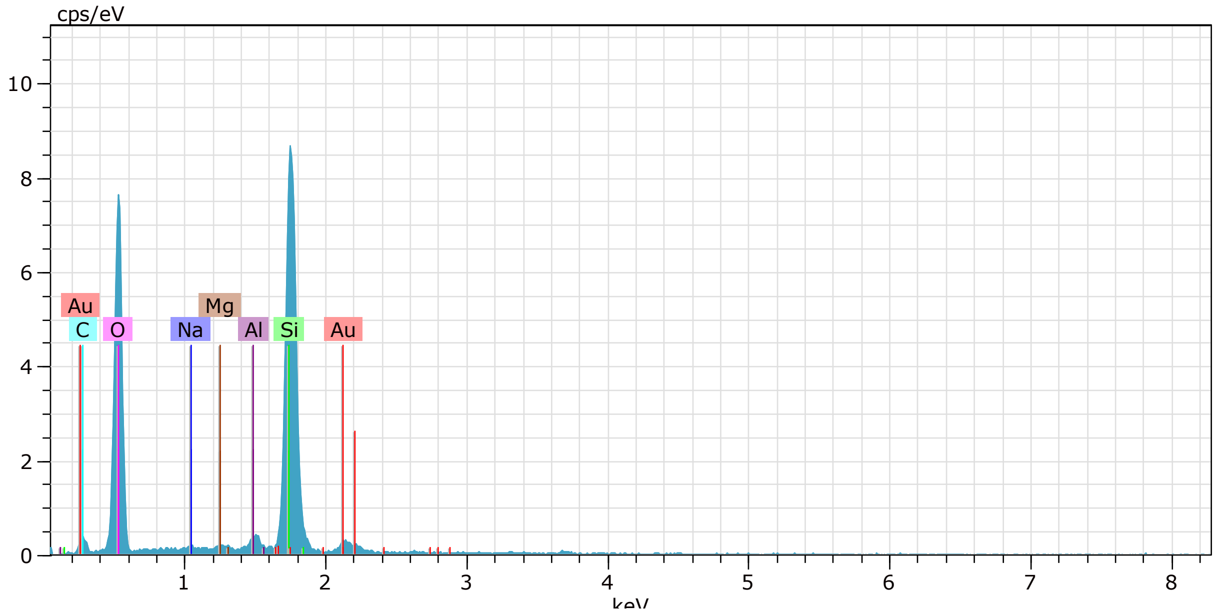

Reference

Han, X., Suess, E., Sahling, H. & Wallmann, K. Fluid venting activity on the Costa Rica margin: new results from authigenic carbonates. *Int. J. Earth Sci.* **93**, 595–611 (2004).
